# First case of *Plasmodium knowlesi* infection in a Japanese traveller returning from Malaysia

**DOI:** 10.1186/1475-2875-12-128

**Published:** 2013-04-15

**Authors:** Ryutaro Tanizaki, Mugen Ujiie, Yasuyuki Kato, Moritoshi Iwagami, Aki Hashimoto, Satoshi Kutsuna, Nozomi Takeshita, Kyoko Hayakawa, Shuzo Kanagawa, Shigeyuki Kano, Norio Ohmagari

**Affiliations:** 1 Disease Control and Prevention Center, National Center for Global Health and Medicine, 1-21-1 Toyama, Shinjuku-ku, Tokyo, 162-8655, Japan; 2 Department of Tropical Medicine and Malaria, Research Institute, National Center for Global Health and Medicine, 1-21-1 Toyama, Shinjuku-ku, Tokyo, 162-8655, Japan

**Keywords:** Plasmodium knowlesi, Malaria, Traveller, Imported, Mefloquine, Japan

## Abstract

This is the first case of *Plasmodium knowlesi* infection in a Japanese traveller returning from Malaysia. In September 2012, a previously healthy 35-year-old Japanese man presented to National Center for Global Health and Medicine in Tokyo with a two-day history of daily fever, mild headaches and mild arthralgia. Malaria parasites were found in the Giemsa-stained thin blood smear, which showed band forms similar to *Plasmodium malariae*. Although a nested PCR showed the amplification of the primer of *Plasmodium vivax* and *Plasmodium knowlesi*, he was finally diagnosed with *P. knowlesi* mono-infection by DNA sequencing. He was treated with mefloquine, and recovered without any complications. DNA sequencing of the PCR products is indispensable to confirm *P. knowlesi* infection, however there is limited access to DNA sequencing procedures in endemic areas. The extent of *P. knowlesi* transmission in Asia has not been clearly defined. There is limited availability of diagnostic tests and routine surveillance system for reporting an accurate diagnosis in the Asian endemic regions. Thus, reporting accurately diagnosed cases of *P. knowlesi* infection in travellers would be important for assessing the true nature of this emerging human infection.

## Background

The first naturally acquired zoonotic infection with *Plasmodium knowlesi* in a human was reported in 1965 [1]; thereafter, no such infections were reported for almost 40 years. In 2004, however, Singh *et al.*[2] reported that natural *P. knowlesi* infections in humans were common parasite species in Malaysia. Cox-Singh *et al.*[3] subsequently described that *P. knowlesi* infections in humans were observed throughout wide areas of Southeast Asia, including Thailand, Myanmar, the Philippines, Singapore and Indonesia. Additionally, 12 cases of *P. knowlesi* infection in travellers have been reported from non-malaria endemic countries thus far [1,4-14]. Here, this is the first confirmed case of *P. knowlesi* infection in a Japanese traveller.

## Case presentation

**Figure 1:**
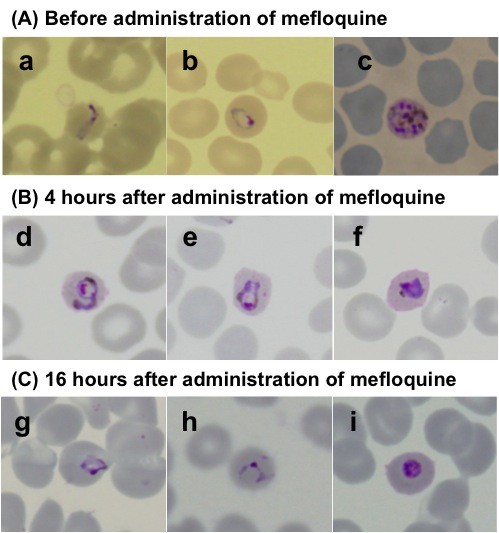
**Microscopic analysis of Giemsa-stained thin blood-smears from a patient infected with *Plasmodium knowlesi *malaria parasites.** The blood smears were taken before (**A**), four hours after (**B**), and 16 hours after treatment (**C**), respectively. **A**) Before mefloquine treatment the following blood-stage parasites were observed: a) band form, b) ring form, and c) schizont. **B**, **C**) Post-mefloquine treatment (**B**, **C**), the ring forms (d, e, g, h) and trophozoites (f, i) gradually changed into irregular forms. The parasitaemias calculated from slides **A**, **B**, and **C** were 0.2%, 0.05%, and 0.013%, respectively.

**Figure 2:**
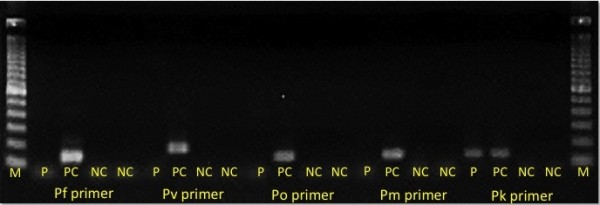
**Nested PCR results from the inner primers.** The region amplified was a partial sequence of the cytochrome *b* gene (mitochondrial DNA) from different malaria parasite species (~130-180 bp). M: DNA size marker, P: patient, Pf: *Plasmodium falciparum*, Pv: *Plasmodium vivax*, Po: *Plasmodium ovale*, Pm: *Plasmodium malariae* and, Pk: *Plasmodium knowlesi*. PC: positive control, NC: negative control. Forward primers for the outer PCR and the inner PCR were the same as those reported by Putaporntip *et al.*[15]. The sequences of the reverse primers used for the outer PCR and the inner PCR are as follows: PCBR-ed (5^′^-ACATAATTATAACCTTACGGTCTG-3^′^), PfCBR-ed (5^′^-GATTTGTTCCGCTCAATAC-3^′^), PvCBR-ed (5^′^-CTGTATTGTTCTGCTCAA-3^′^), PoCBR-ed (5^′^-CTGTATTGTTCTGCTCAT-3^′^), PmCBR-ed (5^′^-CTGTATTGTTCTGCACAG-3^′^), PkCBR-ed (5^′^-GTATTGTTCTAATCAGTGTA-3^′^). Nested PCR primers for the small-subunit rRNA from parasite nuclear DNA were the same as those reported by Kimura *et al.*[16] (gel not shown). The inner PCR primer (reverse) used to amplify *P. knowlesi* DNA (SS-rRNA-Pk-R) had the following sequence: 5^′^-AAGAGTTCTAATCTCCGGAGAGAAAAG-3^′^. DNA sequences of the partial cytochrome *b* gene and SSU rRNA gene were deposited in the DNA Data Bank of Japan (DDBJ). The DDBJ accession numbers of the DNA sequences of the partial cytochrome *b* gene and SSU rRNA gene were AB787188 and AB787187, respectively. A positive band for the patient^′^s blood sample with *P. knowlesi* primer sets was shown.

In September 2012, a previously healthy 35-year-old Japanese man presented to the travel clinic in National Center for Global Health and Medicine (NCGM), Tokyo with a two-day history of daily fever, mild headache, and mild arthralgia. He had visited Malaysia for entomological and botanical field investigations over a two-month period and had stayed at Temengor (four weeks), Johor (two weeks), and Kuala Lumpur (two weeks). While in Temengor, he stayed in a tent located near a forest and had not used any malaria prevention measures, such as bed nets, mosquito repellents or chemoprophylaxis. During his stay, he was bitten by mosquitoes and saw some wild monkeys. He had no health problems and was in a good physical condition until he experienced a sudden high fever (39.0°C axillary temperature) the day after his return to Japan. He had fever spikes of >38.0°C in a 24-hour period. On the third day of his illness, he was admitted to NCGM. On admission his body temperature was 37.0°C, with blood pressure of 111/80 mmHg, pulse rate of 115 beats per minute, respiration rate of 16 per minute, and oxygen saturation of 99% (room air). Physical examination revealed mild splenomegaly and left upper abdominal pain. Laboratory investigations showed thrombocytopenia (47 × 10^3^ cells/mm^3^, reference range 150–350 cells/mm^3^), elevated liver enzymes (serum aspartate aminotransferase 49 U/L, reference range 13–33 U/L; alanine aminotransferase 41 U/L, reference range 8–42 U/L; alkaline phosphatase 428 U/L, reference range 115–359 U/L; and total bilirubin 1.1 mg/dl, reference range 0.3-1.2 mg/dl), a high C-reactive protein level (11.59 mg/dl, reference range 0–0.3 mg/dl), no anaemia (haemoglobin 17.3 g/dl, reference range 13.5-17.0 g/dl), and a normal leukocyte count (3,860 cells/mm^3^, reference range 3,500-8,500/mm^3^). The patient’s free blood glucose levels and renal function were normal. Although rapid diagnostic tests (BinaxNOW Malaria®; Alere, Inc, Scarborough, USA) for the *Plasmodium falciparum* histidine-rich protein 2 and pan-malarial aldolase were negative, malaria parasites were identified in the patient’s Giemsa-stained thin blood smear from which a parasitaemia of 0.2% (10,120 parasites/μl) was calculated. The morphological features of the parasites comprised band forms, which are similar to those characteristics of *Plasmodium malariae,* and heavily pigmented schizonts inside non-enlarged erythrocytes (Figure 1). To make a definitive diagnosis, two partial gene regions (the small subunit rRNA from nuclear DNA and cytochrome *b* from mitochondrial DNA) from five malaria parasite species were tried to be amplified by nested PCR, using respective primer sets for *P*. *falciparum*, *Plasmodium vivax*, *Plasmodium ovale*, *P*. *malariae* and *P. knowlesi*. The positive result was obtained by the primer sets of *P. knowlesi* and *P. vivax* in the amplification of the partial small subunit rRNA gene. However, in the amplification of the partial cytochrome *b* gene, the positive result was obtained by the *P. knowlesi* primer sets (Figure 2). The sequences of the PCR products were determined using an ABI 3130*xl* Genetic Analyzer (Applied Biosystems, CA, USA) after TA cloning. From this sequence analysis, the patient was confirmed that even the PCR product amplified by the *P. vivax* primer sets for the partial small subunit rRNA gene was proved to be the DNA of *P. knowlesi*. He was treated with mefloquine (1,500 mg base), then was repeated the microscopic screening for parasites every 12 hours for confirming parasite disappeared. The clinical course of the mefloquine treatment in this patient was as follows: the initial band-form and schizont-stage parasites seen in the Giemsa-stained blood smears disappeared six hours after administration of the drug and, gradually, the shape of the ring-form parasites became more irregular over time (Figure 1). The parasites disappeared completely from the blood 40 hours later, and the patient’s fever resolved 28 hours after mefloquine administration. The patient was discharged from hospital on the seventh day post-admission, without any complications. No relapses or any other health problems were observed over a five-month period.

## Conclusions

Most *P. knowlesi* infections are reported in the rural jungle areas of the Malaysia peninsular and in Borneo (Sabah [17] and Sarawak [2] province) where both of the infectious hosts (macaque and leaf monkeys) and vectors (*Anopheles* mosquitoes) predominantly exist. The patient in this case was probably infected with *P. knowlesi* in the Temengor jungle area as he had not entered any other areas of jungle for four weeks before returning to Japan. Interestingly, the incubation period in this patient was probably over four weeks, which is longer than the nine to 12 day incubation period reported for *P. knowlesi*[18]. This finding is consistent with a case report for a different traveller who also experienced a long incubation period (>17 days) [13]. Clinical data for *P. knowlesi* infection in travellers are limited; therefore, more data are needed to determine the correct incubation period for *P. knowlesi*.

No cases of treatment failure have been reported in travellers treated with anti-malarial drugs such as quinine [4], doxycycline [4], mefloquine [8], atovaquone/proguanil [5,10,14], and artemether/lumefantrine [12]. In the present case, mefloquine enabled the patient to recover without any complications. Chloroquine has been usually used for uncomplicated *P. knowlesi* infection [19]; however, in recent years, a prospective comparative study suggested that early intravenous artemisinin treatment improved the prognosis in severe cases of *P. knowlesi* infection [17]. The major risk factors of increasing severity are associated with a high parasitaemia [17,20] and thrombocytopenia [20]. The risk factors of severity in *P. knowlesi* infection should be evaluated appropriately, and if necessary, should be considered the intravenous artemisinin treatment as other malaria parasite species [21].

Microscopic examination of Giemsa-stained blood smears is the gold standard for differentiating *Plasmodium* species; however, differentiating *P. knowlesi* from other malaria species is very difficult because *P. knowlesi* band forms and schizonts of mature trophozoites in *P. knowlesi* are similar to those of *P. malariae*, while the ring forms of *P. knowlesi* early trophozoites are similar to those of *P. falciparum*[2,17,18]. This patient was initially suspected of contracting *P. malariae* after observing his Giemsa-stained thin blood smear (Figure 1). Although PCR is required to obtain a definitive diagnosis, the spurious amplification of a *P. vivax* gene using *P. knowlesi*-specific primers has been reported [22]. In the present study, on the other hand, a *P. knowlesi* gene was amplified using *P. vivax*-specific primers [16]. These spurious amplifications were observed when the target gene of PCR was the small subunit rRNA gene [22] because of the similarity between the DNA sequences of *P. knowlesi* and *P. vivax*. Thus, a caution is needed for using this gene to differentiate malaria species by PCR. Indeed, some cases of *P. knowlesi* infection in travellers have been initially diagnosed as *P. ovale*[4] or *P. vivax*[13] by nested PCR; however, these were later confirmed as *P. knowlesi* by DNA sequencing. Therefore, DNA sequencing is indispensable for final confirmation of *P. knowlesi* infection.

There is limited access to DNA sequencing procedures for identifying *P. knowlesi* in endemic areas. Hence, accurate reporting of clinical and epidemiological data should contribute to better understanding of the clinical features of *P. knowlesi* infection, as well as the true incidence of the disease. Close monitoring of febrile travellers returning from *P. knowlesi*-endemic areas and complete travel histories (including exposure to areas with wild monkeys) should prompt suspicion of *P. knowlesi* infection [17] and a move towards further diagnostic tests. Additionally, greater awareness of the risk of the emerging *P. knowlesi* problem in travellers is necessary among health care workers in non-endemic countries. Thus, reporting accurately diagnosed cases of *P. knowlesi* infection in travellers would be important for assessing the true nature of this emerging human infection.

## Consent

Oral informed consent was obtained from the patient for publication of this case report and any accompanying images after explanation of the report objectives.

## Competing interests

All authors declare that they have no competing interests.

## Authors’ contributions

RT, MU, MI, YK, and S Kano wrote the paper. RT, MU, AH, S Kutsuna, NT, KH, S Kanagawa and NO were the physicians responsible for the patient. MU, YK, MI, S Kano supervised molecular characterization of parasite, conceived the study, its design and coordination. MI and S Kano performed microscopic examination of the blood smears, DNA analyse and drafted the manuscript. All authors have read and approved the final manuscript.
